# Traditional invasive vs. minimally invasive esophagectomy: a multi-center, randomized trial (TIME-trial)

**DOI:** 10.1186/1471-2482-11-2

**Published:** 2011-01-12

**Authors:** Surya SAY Biere, Kirsten W Maas, Luigi Bonavina, Josep Roig Garcia, Mark I van Berge Henegouwen, Camiel Rosman, Meindert N Sosef, Elly SM de Lange, H Jaap Bonjer, Miguel A Cuesta, Donald L van der Peet

**Affiliations:** 1 Department of Surgery, VU university medical center, Amsterdam, the Netherlands; 2 Department of Clinical Epidemiology and Biostatistics, VU university medical center, Amsterdam, the Netherlands; 3 Department of Surgery, I.R.C.C.S. Policlinico San Donato, University of Milan, Milan, Italy; 4 Department of Surgery, Hospital Universitari de Girona Dr Josep Trueta, Girona, Spain; 5 Department of Surgery, Academic Medical Center, Amsterdam, the Netherlands; 6 Department of Surgery, Canisius Wilhelmina Hospital, Nijmegen, the Netherlands; 7 Department of Surgery, Atrium Medical Center, Heerlen, the Netherlands

## Abstract

**Background:**

There is a rise in incidence of esophageal carcinoma due to increasing incidence of adenocarcinoma. Probably the only curative option to date is the use of neoadjuvant therapy followed by surgical resection. Traditional open esophageal resection is associated with a high morbidity and mortality rate. Furthermore, this approach involves long intensive care unit stay, in-hospital stay and long recovery period. Minimally invasive esophagectomy could reduce the morbidity and accelerate the post-operative recovery.

**Methods/Design:**

Comparison between traditional open and minimally invasive esophagectomy in a multi-center, randomized trial. Patients with a resectable intrathoracic esophageal carcinoma, including the gastro-esophageal junction tumors (Siewert I) are eligible for inclusion. Prior thoracic surgery and cervical esophageal carcinoma are indications for exclusion. The surgical technique involves a right thoracotomy with lung blockade and laparotomy either with a cervical or thoracic anastomosis for the traditional group. The minimally invasive procedure involves a right thoracoscopy in prone position with a single lumen tube and laparoscopy either with a cervical or thoracic anastomosis. All patients in both groups will undergo identical pre-operative and post-operative protocol. Primary endpoint of this study are post-operative respiratory complications within the first two post-operative weeks confirmed by clinical, radiological and sputum culture data. Secondary endpoints are the operative data, the post-operative data and oncological data such as quality of the specimen and survival. Operative data include duration of the operation, blood loss and conversion to open procedure. Post-operative data include morbidity (major and minor), quality of life tests and hospital stay.

Based on current literature and the experience of all participating centers, an incidence of pulmonary complications for 57% in the traditional arm and 29% in the minimally invasive arm, it is estimated that per arm 48 patients are needed. This is based on a two-sided significance level (alpha) of 0.05 and a power of 0.80. Knowing that approximately 20% of the patients will be excluded, we will randomize 60 patients per arm.

**Discussion:**

The TIME-trial is a prospective, multi-center, randomized study to define the role of minimally invasive esophageal resection in patients with resectable intrathoracic and junction esophageal cancer.

**Trial registration (Netherlands Trial Register):**

NTR2452

## Background

The incidence of esophageal cancer is increasing in the Western world. In the Netherlands, in the year 1990 some 807 patients were diagnosed with esophageal cancer, whereas in 2005, this number reached a staggering 1546 [1]. It is expected that this rise in incidence will continue in the years to come. This substantial increase in incidence can be accounted for by an increase in the number of adenocarcinomas diagnosed.

Approximately one third of the patients are considered candidates for a curative approach. Surgical resection with radical lymphadenectomy, usually after neoadjuvant chemotherapy or chemo-radiotherapy, remains the only curative option for resectable esophageal cancer. Surgery is considered when the tumor is staged as cT1-3 N0-1 M0. Despite the curative intent, some 30% of all resections have microscopically residual disease (R_1_). Most patients present with stage III esophageal cancer, which has a 5-year survival of approximately 20-25% [2]. In addition, the possible value of neoadjuvant chemoradiotherapy or chemotherapy is currently being investigated. However, a meta-analysis by Gebski et al. has shown that surgery following chemoradiotherapy for both squamous cell carcinoma and adenocarcinoma has a survival benefit of 13% after 2 years. For neoadjuvant chemotherapy this survival benefit was 7% after 2 years for adenocarcinomas [3].

The three main surgical approaches utilized worldwide for intrathoracic esophageal cancer are the following: (1) the three stage transthoracic resection (i.e. right postero-lateral thoracotomy, laparotomy and cervicotomy) with a cervical anastomosis; (2) the two stage transthoracic resection (i.e. laparotomy, and right postero-lateral thoracotomy, including the Ivor Lewis approach with an intrathoracic anastomosis); and (3) the two stage transhiatal resection (i.e. laparotomy and cervicotomy with cervical anastomosis) [4]. Transhiatal esophagectomy according to Orringer is generally performed for gastro-esophageal junction cancers [5]. Nevertheless, cancer of the lower esophagus metastasized, according to the Tumor-Node-Metastasis classification, in more than 45% to the lymph nodes in mediastinum and carina. Therefore patients with reasonable general condition are increasingly surgically approached transthoracically. In the randomized study by Hulscher et al. and long term follow-up, comparing the transhiatal and transthoracic esophageal resection, an important trend of better survival has been observed in the transthoracically approached patients [6,7]. This transthoracic procedure is associated with a high morbidity and mortality rate of approximately 50-70% and 5% respectively [6]. Moreover, the extensive nature of this open approach has a significant negative impact on the quality of life of these patients and is associated with a long in-hospital recovery.

Minimally invasive esophageal (MIE) resection for cancer avoiding the thoracotomy and laparotomy can reduce the amount of trauma of the required surgery with the same oncological value. This will imply a reduction of the post-operative morbidity, a shortening of the recovery time and an increase of quality of life. Evidence of the short term benefits of minimally invasive surgery over open procedures with similar oncological outcome is accumulating. Less perioperative complications, shorter hospital stay and faster post-operative recovery appear to be the main advantages. MIE involves a right thoracoscopy and laparoscopy, either with a cervical or intrathoracic anastomosis. The thoracic phase of this procedure can be performed through a lateral right thoracic approach with a right lung block by selective intubation or in prone position without selective lung block. This prone approach, with partial lung collapse, will result in lower percentage of pulmonary complications [8,9].

To date, no randomized trials have been performed comparing any modality of minimally invasive esophagectomy with an open traditional approach [10]. Given the values of postoperative morbidity, quality of life and quality of the specimen, the aim of this prospective randomized study is to compare the MIE by right thoracoscopy in prone position and laparoscopy with the open esophageal resection by right thoracotomy and laparotomy in left lateral decubitus, for those patients possessing intrathoracic resectable esophageal cancer. This comparison will provide further evidence supporting the minimally invasive and cost-effective approach for esophageal cancer.

## Methods

### Study objectives

The TIME trial is prospective, multi-center, randomized study comparing traditional transthoracic esophageal resection with minimally invasive resection for esophageal cancer. Patients with resectable intrathoracic esophageal cancer are randomized for either (a) minimally invasive transthoracic esophageal resection in prone position or (b) traditional open transthoracic esophageal resection. Our hypothesis is that patients undergoing a minimally invasive esophagectomy have fewer morbidity, a shorter duration of the intensive care unit (ICU) admission and a better quality of life than following the traditional approach.

### Endpoints

The primary endpoint of this study concerns the respiratory complications (i.e. infections) within two weeks after the operation. This is categorized as: grade 1) initial respiratory distress after operation with continuation of mechanical ventilation; grade 2) after successful detubation, clinical manifestation of respiratory infection caused by (broncho) pneumonia, confirmed by thorax X-ray or CT scan of the thorax and a positive sputum culture; and grade 3) other thoracic infections like post-operative empyema either caused or not by leakage from the gastric conduit necessitating drainage or reoperation [modified from Hulzebos et al., [11]]. Consequences for patients range from extensive physiotherapy, involving oxygen and specific antibiotics to intubation and mechanical ventilation. Furthermore, important respiratory deterioration after extubation, involving reintubation and mechanical ventilation will lead to the necessity of a CT scan of the thorax and abdomen, and thus endoscopic examination of the gastric tube and anastomosis in order to rule out a leakage.

The secondary endpoints are operation related events (e.g. duration of operation, blood-loss and conversion to open procedure in MIE group) and re-operations. Moreover, general morbidity (major and minor) is recorded. Minor complications are defined as wound infections, venous thrombosis or other. Major complications consist of- apart from respiratory complications- post-operative bleeding, anastomosis leakage, mediastinitis, and re-operations within the in-hospital period. Furthermore, post-operative recovery data are length of ICU and hospital stay (days), type and number of analgesics needed after operation, VAS-pain-score, return to fluid and normal diet, quality of life questionnaires (SF-36 and EORTC QLQ-OES18) [12], and quality of the specimen resected (length of specimen, number and location of lymph nodes resected, and circumferential resection margin). Also hospital mortality and readmissions are recorded. Furthermore survival will be analyzed.

### Power of the study

According to the published literature and own experience at the VU university medical center a difference in respiratory infections of 28% is found between the traditional open procedure (57%) and the MIE procedure (29%) [6,7,9,13-15]. To demonstrate this difference of 28%, using a alpha = 0.05 and beta = 0.80, two groups of 48 patients are needed. This is based on a two-sided significance level (alpha) of 0.05 and a power of 0.80. Estimating that approximately 20% of the patients may be excluded, 60 patients will be randomized per arm.

### Inclusion criteria

Candidates to be included in this study are all patients with a histologically proven squamous cell carcinoma, adenocarcinoma or undifferentiated carcinoma of the intrathoracic esophagus and Siewert I junction tumors which are surgically resectable (T1-3, N0-1, M0) and treated by neoadjuvant therapy. The age of the patients must be ≥ 18 and ≤ 75 years. Moreover, the included patients must have a European Clinical Oncology Group (ECOG) performance status of 0, 1 or 2; and their written informed consent is obligatory.

### Exclusion criteria

Patients are excluded as subjects if there is a carcinoma of the cervical esophagus or have undergone prior thoracic surgery or no informed consent is provided. An exclusion list is maintained by all participating centers in order to analyze the quality of the randomization rate.

### Participating surgeons and clinics

To prevent surgeon bias, the open and laparoscopic operations have to be performed by experienced surgeons in conventional esophageal resections with experience of at least 10 minimally invasive esophagectomies. Duration of operation, conversion to open surgery, and complication rate may be related as factors to experience, yearly volume and the learning curve of the participating surgeon [16]. The surgeons in the three Dutch centers have been proctored by the two experienced minimally invasive surgeons of the VU university medical center. After the fist five patients operated on by an combined team, the video's of the last two of a series of 15 patients who had undergone a minimally invasive approach were examined by the VU university medical center surgeons. Only the surgeons with sufficient experience and skill after the proctoring series are allowed in participation in the trial. The surgeons of the two other centers are already well experienced in minimally invasive esophagectomy. In order to prevent institution bias, only hospitals with high volume (>20 esophagectomies/year) will participate in this trial.

Six European academic and non-academic centers will participate in the study: Academic Medical Center, Amsterdam, the Netherlands; Atrium Medical Center, Heerlen, the Netherlands; Canisius Wilhelmina Ziekenhuis, Nijmegen, the Netherlands; Hospital Universitari de Girona, Dr Josep Trueta, Girona, Spain; I.R.C.C.S. Policlinico San Donato, Milan, Italy; and VU university medical center (Vumc), Amsterdam, the Netherlands.

### Randomization

**Figure 1:**
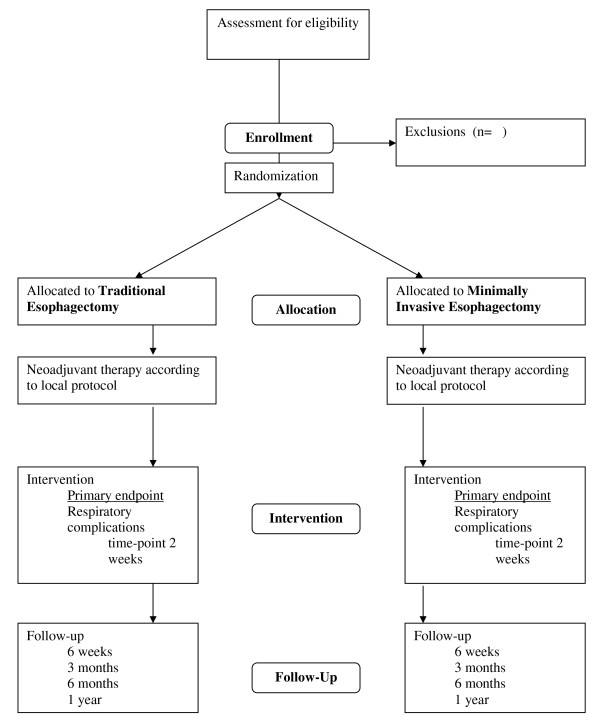
**TIME-trial flowchart**.

The patient will be informed about the trial at the outpatient clinic. When informed consent is obtained, the patient will be randomized at the outpatient clinic. Randomization is performed per center by an internet randomization module maintained by coordinators at the VUmc. As some heterogeneity is expected, e.g. difference in type of neoadjuvant therapy protocol, randomization will be stratified for each center. A flowchart of the study protocol is seen in Figure 1.

### Data collection and statistics

Data are transcribed via datasheets on paper and sent to the VUmc by surface mail. Data are collected daily until the day of discharge. The quality of life questionnaires (SF-36 and EORTC-OES18) are completed by the patient starting preoperatively and post-operatively at 6 weeks, 3 months, 6 months, and at 1 year. There will be regular contact between the study-coordinators and the participating centers. One research fellow will monitor the data of all included patients. Using a SPSS database containing all required parameters, data analysis will be performed in accordance with the intention-to-treat principle, additional per-protocol analysis will also be performed. Groups are, where appropriate, compared using an Independent Samples T-test, otherwise a Wilcoxon test, or Chi-square test. Pain scores will be analyzed using repeated measures analysis.

### Ethics

This study is conducted in accordance with the principles of the Declaration of Helsinki and 'good clinical practice' guidelines. The independent medical ethics committees of the participating centers have approved the study protocol. Prior to randomization, written informed consent will need to be obtained from all patients.

## Surgical Technique

### Pre-operative regimen

Pre-operative preparation has the aim of keeping patients physically and mentally as optimal as possible during the whole period of pre-operative period. Complete information about the diagnostic phase, randomization, neoadjuvant therapy and surgical intervention is very important here. Coordination of this preparation period is undertaken by a research nurse, the study-coordinator and the participating surgeons.

All patients will have regular consultations by a dietician during the whole pre-operative path. Supplemental nutritional feeding can be initiated and if necessary a thin nasogastric tube can be placed for feeding purposes. Also, all patients will be consulted by a physiotherapist for exercises with emphasis on respiratory improvement. If necessary, a psychological consultation will be arranged.

### Neoadjuvant therapy

All patients included will pre-operatively receive neoadjuvant therapy: chemoradiotherapy or chemotherapy alone according to local protocol. Each center will treat patients, either in category open operation or MIE, in the same way.

### Esophageal resection

The open operation as well MIOE operation consists of a two-field esophageal resection with gastric tube formation followed by cervical or thoracic anastomosis.

For patients undergoing a thoracotomy, a high epidural catheter and a double tube are placed for selective intubation.

### Traditional transthoracic esophagectomy

In the open group a three-stage procedure is followed. After selective intubation to block the right lung, the patient is placed in a left lateral decubitus position. The first stage is started with a right posterolateral thoracotomy. The esophagus and its overlying mediastinal pleura is mobilized with mediastinal and carina lymphadenectomy. For the second stage, the patient is turned to a supine position. Through a supra-umbilical laparotomy the stomach is mobilized with special care of the gastro-epiploic vessels and a lymphadenectomy of the celiac trunk is performed. The dissection finalizes at the hiatus with anterior extension and careful dissection of the gastro-esophageal junction along the planes. For the last stage a cervical incision is made and the esophagus dissected free. Retrieval of the specimen through the laparotomy wound is performed and a gastric conduit created. No pyloroplasty is usually performed. A jejunostomy catheter is placed for feeding purposes. A gastric tube-esophageal anastomosis is then established in an end-to-side fashion.

If an thoracic anastomosis is made, the first phase of it commences with an abdominal approach involving the patient in supine position. The second phase (thoracic) is performed with the patient in a left decubitus position. The retrieval of the specimen will be achieved through the thoracotomy wound. The anastomosis will be made high in the thorax, proximal at the level of the divided vena azygos.

### Minimally invasive transthoracic esophagectomy

The MIE also has a three-stage procedure. The difference from the procedure of the open operation is that there is no need for selective intubation with the exception of patients in whom an thoracic anastomosis is planned (a Fogarty balloon catheter is placed under bronchoscopy view in the right primary bronchus and inflated only during the anastomosis phase). After anesthesia, the patient is turned to a prone position. Four trocars are placed along the medial edge of the scapula. Modest insufflation using CO2 will raise the intrathoracic pressure between 6 and 8 mmHg. Radical esophagectomy is performed along the pericard sac, pulmonal veins, right bronchus, aorta resecting the esophagus with the mediastinal pleura and lymphadenectomy (peri-esophageal, lower posterior mediastinal, carina and right paratracheal). After completion of this phase the thorax is drained and the trocar sites are closed.

The patient is then placed in a supine position. After introduction of four trocars the mobilization of the stomach is performed similar to the traditional procedure (with paracardiac left and right, lesser and greater curvature and celiac trunc lymphadenectomy). After dissection of the esophagus at cervical level, the specimen is retrieved through a well protected trans-umbilical mini-laparotomy (6-8 cm). The esophageal-proximal stomach resection is performed extra-corporeally and a small gastric conduit (3-4-cm) created, then conducted to the cervical wound and there anastomosed. A jejunostomy catheter is placed.

If a thoracic anastomosis is made (two phase procedure), the first phase commences with a laparoscopy involving the patient in supine position. The only difference with the three stage MIE (abovementioned) is the laparoscopic creation of the gastric tube and hiatal dissection. A jejunostomy catheter is placed. The second phase (thoracic) is performed with the patient in prone decubitus position. The esophagus is dissected free up to the distal trachea, the azygos vein is divided and lymphadenectomy performed. After division of the esophagus, a purse string is placed at the proximal esophagus. A posterior mini-thoracotomy (6 cm) is performed, the lung is blocked, and a 25 circular stapler anvil placed in the proximal esophagus. The specimen is retrieved, resected and through it the circular stapler is placed and an end-to-side anastomosis performed. The rest of the loop is resected by an endoscopic stapler and the thoracic cavity drained.

### Post-operative management

Patients in both groups will receive similar post-operative treatment. All patients will after surgery be admitted intubated at the intensive care unit (ICU). After stabilization and detubation, the patient will if indicated be admitted to the general surgical ward or to the medium care unit (MCU). In the first days after surgery analgesics are administered by the epidural route. In the event of epidural failure, post-operative pain will be treated intravenously by a 'patient controlled analgesia' (PCA); when necessary through a pump with morphine. Patients will be instructed about the PCA pump by an anesthesiologist and morphine doses will be noted. Patients will have a nasogastric tube in-situ for at least 5 days as some gastric conduit distension is expected. All patients will receive postoperative physiotherapy for breathing-exercises the day after surgery. To regain early mobilization, starting day 1, patients are encouraged to sit out of bed in the general surgical ward. Enteral feeding is commenced day 1 after operation through the jejunostomy and increased to optimal feeding at day 3. At day 5, after gastrographine swallow X ray, the nasogastric tube is retired and started with liquids. Normal diet can be progressively resumed while jejunostomy feeding is decreased. Patients will be discharged when they are able to eat normal food, can walk and are comfortable with oral analgesia. Delay to "social" reasons will be noted. Completion of the feeding over the jejunostomy may be continued after discharge. Patient follow-ups are carried out at the outpatient clinic at 6 weeks, 3 months, 6 months and 1 year after discharge. During these visits, the quality of life questionnaires (SF-36 and EORTC QLQ-OES18) will be completed. The regular follow-up will continue up to 5 years after surgery.

## Discussion

Surgery on cancer of the esophagus is considered to be one of the most extensive and traumatic oncological surgical procedures. Open resection not only involves a long operation time and large incisions but also necessitates post-operative care in the intensive care unit, a long in-hospital recovery with decreased quality of life and carries a significant risk of morbidity and death.

MIE can reduce the post-operative morbidity, in particular the respiratory complications which are most encountered. Different landmark studies have reported significantly low pulmonary complications rates using the minimally invasive transthoracic approach. Palanivelu et al. report in their minimally invasive series of 130 patients in prone-position, 2.3% pulmonary complications [9] whereas Luketich et al. report in their series of 222 patients in left lateral decubitus MIE, 18% pulmonary complications [13]. Other authors report a similar incidence in pulmonary complications with probably a slight advantage for the thoracoscopic resection in prone position, being in the experience of the VUmc around 25%. In contrast, Hulscher et al. observed 57% pulmonary complications in patients undergoing the traditional three-stage transthoracic esophagectomy [6]. Furthermore, median length of ICU stay was 1 day in the series of Palanivelu and Luketich whereas in the traditional series of Hulscher the ICU stay was 6 days. Oncologically, the type of resected specimen and lymph nodes are comparable with the open series and disease-free and overall survival reported for MIE and traditional resection are quite comparable. These aforementioned landmark studies favor minimally invasive esophagectomy in terms of pulmonary complications and recovery.

Despite the advantages of the procedure, still only a small percentage of all esophageal resections for cancer are performed minimally invasive. Although, important MIE series have demonstrated feasibility and important short term advantages, yet to date the beneficiary effects of minimally invasive esophagectomy have not been proven by a randomized trial [10]. Therefore, a randomized comparison between traditional esophagectomy and minimally invasive esophagectomy is necessary. This randomized trial can provide further evidence supporting the minimally invasive and cost-effective approach for esophageal cancer.

## Abbreviations

VAS-pain score: Visual Analogue Scale Pain score

## Competing interests

The authors declare that they have no competing interests.

## Authors' contributions

SSAYB and KWM drafted the manuscript. MAC and DvdP designed the protocol and co-authored the writing of the manuscript. ESMdL contributed to design of the protocol and calculation of the power of the study. All other authors participated in the design of the study and are local investigators in the participating centers. All authors were involved in editing the manuscript and approved the final text of the manuscript.

## Pre-publication history

The pre-publication history for this paper can be accessed here:

http://www.biomedcentral.com/1471-2482/11/2/prepub

## References

[B1] Netherlands Cancer Registry Incidentiecijfers oesofaguscarcinomen Integrale Kankercentra http://www.ikcnet.nl/2008-9-26

[B2] Enzinger PC Mayer RJ Esophageal cancer New England Journal of Medicine 2003 349 2241 2252 10.1056/NEJMra035010 14657432

[B3] Gebski V Burmeister B Smithers Foo K Zalcberg J Simes J Survival benefit from neoadjuvant chemoradiotherapy or chemotherapy in oesophageal carcinoma: a meta-analysis Lancet Oncology 2007 8 226 234 10.1016/S1470-2045(07)70039-6 17329193

[B4] Cuesta MA van den Broek WT van der Peet DL Meijer S Minimally invasive esophageal resection Seminars in Laparoscopic Surgery 2004 11 147 160 15510310 10.1177/107155170401100304

[B5] Scheepers JJ Mulder CJ van der Peet DL Meijer S Cuesta MA Minimally invasive oesophageal resection for distal oesophageal cancer; a review of literature Scandinavian Journal of Gastroenterology 2006 41 suppl 123 134 10.1080/00365520600664425 16782631

[B6] Hulscher JBF van Sandwick JW de Boer AG Wijnhoven BP Tijssen JG Fockens P Extended transthoracic resection compared with limited transhiatal resection for adenocarcinoma of the esophagus New England Journal of Medicine 2002 347 1662 1669 10.1056/NEJMoa022343 12444180

[B7] Omloo JM Lagarde SM Hulscher JB Reitsma JB Fockens P van Dekken H Extended transthoracic resection compared with limited transhiatal resection for adenocarcinoma of the mid/distal esophagus: five-year survival of a randomized clinical trial Annals of Surgery 2007 246 992 1000 10.1097/SLA.0b013e31815c4037 18043101

[B8] Cuschieri A Thoracoscopic subtotal oesophagectomy Endoscopic Surgery and Allied Technologies 1994 2 21 25 8081911

[B9] Palanivelu C Prakash A Senthilkumar R Senthilnathan P Parthasarthi R Rajan S Minimally invasive esophagectomy: thoracoscopic mobilization of the esophagus and mediastinal lymphadenectomy in prone position--experience of 130 patients Journal of the American College of Surgeons 2006 203 7 16 10.1016/j.jamcollsurg.2006.03.016 16798482

[B10] Biere SSAY Cuesta MA van der Peet DL Minimally invasive versus open esophagectomy for cancer: a systematic review and meta-analysis Minerva Chirurgica 2009 64 121 133 19365313

[B11] Hulzebos EHJ van Meeteren NLU De Bie RA Dagnelie PC Helders PJM Prediction of postoperative pulmonary complications on the basis of preoperative risk factors in patients who had undergone coronary artery bypass graft surgery Physical Therapy 2003 83 8 16 12495408

[B12] Blazeby JM Conroy T Hammerlid E Fayers P Sezer O Koller M Clinical and psychometric validation of an EORTC questionnaire module, the EORTC QLQ-OES 18 to assess quality of life in patients with oesophageal cancer European Journal of Cancer 2003 39 1384 1394 10.1016/S0959-8049(03)00270-3 12826041

[B13] Luketich JD Alvelo-Rivera M Bunaventura PO Christie NA McCaughan JS Litle VR Minimally invasive esophagectomy: outcomes in 222 patients Annals of Surgery 2003 238 486 494 14530720 10.1097/01.sla.0000089858.40725.68 PMC1360107

[B14] Zingg U McQuinn A DiValentino D Esterman AJ Bessell JR Minimally invasive versus open esophagectomy for patients with esophageal cancer Annals of Thoracic Surgery 2009 87 911 919 10.1016/j.athoracsur.2008.11.060 19231418

[B15] Nguyen NT Hinojosa MW Smith BR Chang KJ Gray J Hoyt D Minimally invasive esophagectomy: lessons learned from 104 operations Annals of Surgery 2008 248 1081 1091 10.1097/SLA.0b013e31818b72b5 19092354

[B16] Birkmeyer JD Stukel TA Siewers AE Goodney PP Wennberg DE Lucas FL Surgeon volume and operative mortality in the United States New England Journal of Medicine 2003 349 2117 2127 10.1056/NEJMsa035205 14645640

